# The Anti-Inflammatory, Analgesic, and Antioxidant Effects of Polyphenols from *Brassica oleracea var. capitata* Extract on Induced Inflammation in Rodents

**DOI:** 10.3390/molecules29153448

**Published:** 2024-07-23

**Authors:** Octavia Sabin, Raluca Maria Pop, Ioana Corina Bocșan, Veronica Sanda Chedea, Floricuța Ranga, Adriana Grozav, Antonia-Mihaela Levai, Anca Dana Buzoianu

**Affiliations:** 1 Pharmacology Toxicology and Clinical Pharmacology, Department of Morphofunctional Sciences, “Iuliu Haţieganu” University of Medicine and Pharmacy, Victor Babeș, No. 8, 400012 Cluj-Napoca, Romania; octavia.sabin@umfcluj.ro (O.S.);; 2 Research Station for Viticulture and Enology Blaj (SCDVV Blaj), 515400 Blaj, Romania; chedeaveronica@yahoo.com; 3 Food Science and Technology, Department of Food Science, University of Agricultural Science and Veterinary Medicine Cluj-Napoca, Calea Mănăștur, No. 3-5, 400372 Cluj-Napoca, Romania; floricutza_ro@yahoo.com; 4 Department of Organic Chemistry, “Iuliu Hațieganu” University of Medicine and Pharmacy, 41 Victor Babeș Street, 400012 Cluj-Napoca, Romania; adriana.ignat@umfcluj.ro; 5 Obstetrics and Gynecology, Department of Mother and Child, “Iuliu Hatieganu” University of Medicine and Pharmacy, Victor Babeș, No. 8, 400012 Cluj-Napoca, Romania; levai.antonia@yahoo.com

**Keywords:** *Brassica oleracea*, polyphenols, anti-inflammatory, analgesic, antioxidant

## Abstract

This study investigates the anti-inflammatory, analgesic, and antioxidant properties of polyphenols extracted from *Brassica oleracea var. capitata* (cabbage) ethanolic extract (BOE). Given the historical use of cabbage in traditional medicine for treating various ailments, this research aims to validate these effects scientifically. The study involved the characterization of BOE’s bioactive compounds using Fourier Transform Infrared Spectroscopy (FTIR) and Liquid Chromatography–Diode Array Detection–Electro-Spray Ionization Mass Spectrometry (HPLC-DAD-ESI MS) analysis. We assessed the anti-inflammatory and analgesic effects of topical and oral BOE administration on rodent models with acute and subacute inflammation. Additionally, the antioxidant capacity of orally administered BOE was evaluated. The results showed that BOE possesses significant levels of phenolic compounds with a potent antioxidant activity. The topical administration of BOE demonstrated notable anti-inflammatory effects in the tested rodent models, which were comparable with nonsteroidal anti-inflammatory drugs. These findings suggest that BOE could be a valuable natural remedy for inflammation-related conditions, supporting its traditional uses and highlighting its potential for further pharmacological development.

## 1. Introduction

Cabbage (*Brassica oleracea var. capitata*) is one of the most cultivated vegetables worldwide. It belongs to the family Cruciferae, which includes cauliflower, broccoli, and kale. Cabbage is consumed raw or processed in different ways, mainly by boiling or fermenting. This popular food has also additional therapeutic uses in traditional medicine. The availability of cabbage in almost all seasons has made it popular in Eastern European traditional medicine [1]. Cabbage is traditionally consumed to ameliorate various respiratory, gastrointestinal, and cardiovascular diseases, and it is included in various diets for diabetic or overweight patients to improve glycemic control or for weight loss [2,3].

The shape of the cabbage leaf makes it easy to apply on the skin in wraps and, in this way, cabbage leaves are used for the treatment of minor skin infections, mastitis, or edema by application of local cataplasms [1]. Topical use is sustained by their antioxidant, anti-inflammatory, antipruritic, and antibacterial properties, which are mainly evaluated in wound-healing studies [1,4,5,6]. The pharmacological properties of cabbage can be explained by its rich composition in bioactive components like glucosinolates, flavonoids, phenols, and anthocyanins (mainly the red version of *Brassica*) [3,5]. Of these, flavonoids and phenolic compounds are associated with an anti-inflammatory effect and an acceleration of skin healing [4]. Although different literature studies report many variations in its experimental use, and differences in the chemical composition given by cultivars’ origin, growth, soil, and climate conditions of cabbage [3,5], its anti-inflammatory, analgesic, and antioxidant effects still need to be proven. Specifically, there are insufficient in vivo investigations on animal models of inflammation that compare topical to oral administration and associate these effects with the plant extract’s composition. Therefore, this study aimed to analyze the basic constituents of *Brassica oleracea var. capitata f. alba* ethanolic extract (BOE) evaluate its anti-inflammatory and analgesic effects after topical and oral administration on two different animal models of inflammation. Second, the study evaluated the antioxidant effect on oral administration of BOE.

## 2. Results

In the present study, *Brassica oleracea* L. *var. capitata* ethanolic extract was characterized to have a complete overview of its composition in bioactive compounds with potential anti-inflammatory and analgesic effects.

### 2.1. BOE Phytochemicals Characterization

#### 2.1.1. Total Polyphenol Content

The total phenolic content (TPC) was estimated spectrophotometrically. The BOE extract had TPC 94.16 ± 4.17 mg GAE equivalents/100 g fresh weight.

#### 2.1.2. Fourier Transform Infrared Spectroscopy (FTIR) Analysis

**Figure 1:**
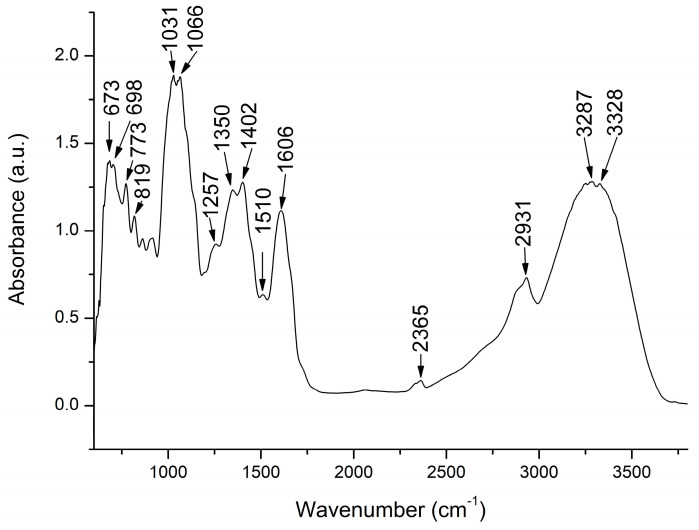
Fourier Transform Infrared Spectroscopy spectrum of *Brassica oleracea* L. *var. capitata* ethanolic extract.

FTIR analysis was performed to offer a general overview concerning the chemical composition of BOE extract. The analysis is based on the vibration rotation movements at a specific frequency of different chemical bonds and functional groups in the analyzed matrices [7]. Accordingly, the FTIR spectrum of the BOE extract is summarized in Figure 1.

The region between 3000 and 4500 cm^−1^ is characterized by the two intense absorption peaks at 3388 cm^−1^ and 3287 cm^−1^, representing O-H stretching vibration that can be attributed to molecules with alcoholic functions, aromatic carbons, or phenolic -OH stretching in the phenolic compounds, respectively [8,9,10]. Next, the region between 1960 and 2850 cm^−1^ was represented by the absorption peak at 2931 cm^−1^ that can be assigned to aliphatic C-H stretching in the methylene -CH^2−^ and methyl -CH^3−^ [3] or to O-H (carboxylic acid) stretching vibration (Li). Next, the fingerprint region is characterized by the appearance of several intense peaks like 1606 cm^−1^, which is attributed to the C=O stretching vibration of lipid esters, or to the C=O and C=C stretching vibrations characteristic of phenolic compounds Also, the peaks between 1650 and 1350 cm^−1^ could be attributed to absorption bands of amino groups or the skeletal bands of aromatic compounds [11]. Thus, the high-intensity peak at 1402 cm^−1^ was attributed to the C-H (Vinyl group) stretching vibration [10] or to aromatic compounds, and the high-intensity peak at 1350 cm^−1^ was attributed to the N=O stretching vibration of nitro compounds [10]. Next, the most intense absorption bands at 1031 cm^−1^ could be assigned to the N=O stretching vibration of nitro compounds [10]. Also, the peak at 1066 cm^−1^ can be attributed to the presence of flavonoids or carbohydrates in the BOE extract [11]. Finally, the peaks between 800 and 650 cm^−1^ could be assigned to the C-H out-of-plane gamma bending modes in aromatic hydrogens [9]. The same specific absorption bands like the O-H in phenols or carboxylic acids, the C-H of aromatic hydrogens, the N=O characteristics for nitro compounds, and S=O in sulfoxide compounds were also identified in the Chinese cabbage [10]. The presence of lucosinolates compounds in the BOE extract can be identified as well due to their specific absorption characteristics, which include the O-H deformation and stretching, the S-H or C-H stretch of methyl groups, and methylene C-H stretching. Overall, glucosinolates have specific absorption bands between 2000 and 2500 cm^−1^ mostly due to N-H, O-H, and C-C bond stretches and between 950 and 1650 cm^−1^, which are regions that have been successfully used to quantify individual or total glucosinolates in broccoli samples [12,13,14].

#### 2.1.3. Liquid Chromatography–Diode Array Detection–Electro-Spray Ionization Mass Spectrometry (HPLC-DAD-ESI MS) Analysis

The LC-MS analysis of BOE extract allowed us to tentatively identify 12 compounds belonging to different classes as presented in Table 1. Namely, there were four desulfo-glucosinolates (desulfo-glucoraphanin, glucoerucin, glucoraphanin, and desulfo-glucobrassicin), three flavonols (kaempherol rhamnoside, isorhamnetin 3-o acetyl glucoside, and quercetin 3-o-6-benzoyl-galactoside), three hydroxycinnamic acid derivatives (hydroxycinnamic acid para coumaric acid, caffeoylquinic acid, and synapoyl glucoside acid) and two flavones (apigenin-glucoside and apigenin-apiosyl-glucoside).

### 2.2. The Effect of BOE on Inflammation

#### 2.2.1. Acute Inflammation Produced by Carrageenan

**Figure 2:**
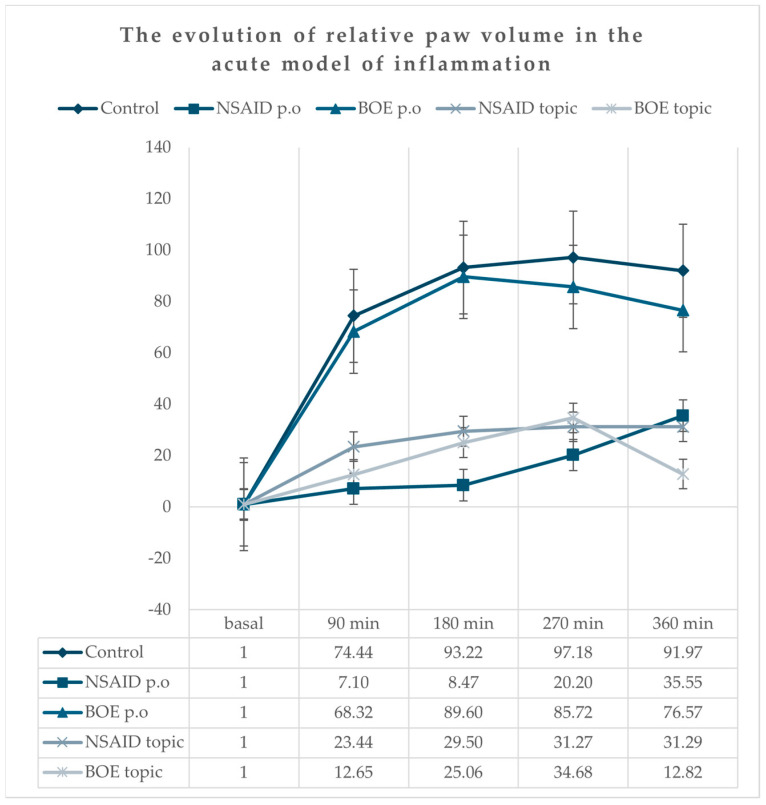
Relative paw edema evolution in time after carrageenan administration (calculated according to the initial paw volume) Legend: NSAID—nonsteroidal anti-inflammatory drug, BOE—*Brassica oleracea* extract, p.o—oral.

All animals presented a marked unilateral peripheral paw edema after carrageenan injection with a progressive increase, reaching the maximum at the 270 min time point (Figure 2). The group treated with diclofenac (NSAID p.o) had the lowest increase in edema compared to the group treated with saline solution (Control) at all time points. The relative paw volume in both the NSAID and BOE topic groups had lower values compared to the relative paw volume in the Control group at all time point measurements without any significant differences when compared to the NSAID p.o group. There was no significant difference between the Control and BOE p.o groups (see Supplementary Table S1).

#### 2.2.2. Subacute Experimental Inflammation

**Figure 3:**
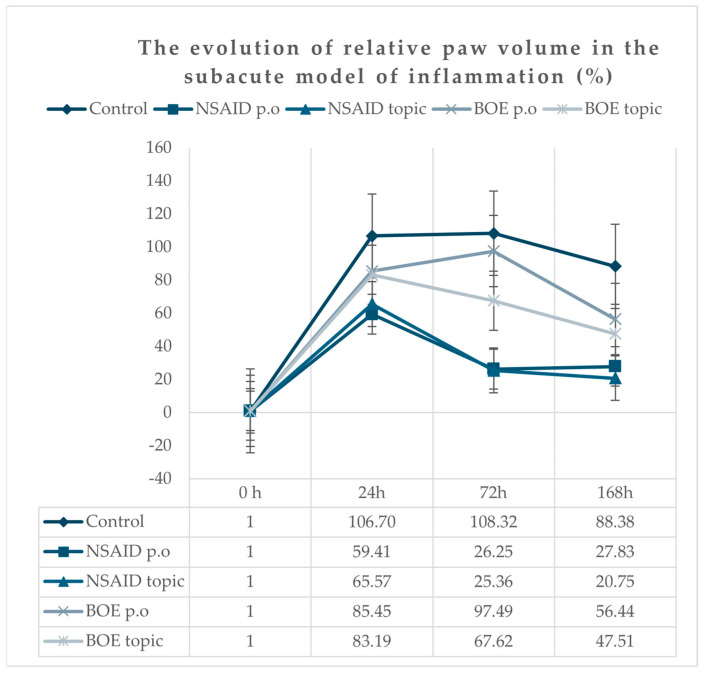
Relative paw inflammation evolution in time after Freund’s adjuvant injection (calculated according to the initial paw volume). Legend: NSAID—nonsteroidal anti-inflammatory drug, BOE—*Brassica oleracea* extract, p.o—oral.

All animals presented a marked unilateral peripheral paw edema after Freund’s adjuvant injection, reaching the maximum at 24 h time point, with a progressive decrease in time. At 168 h after injection, the edema was still present; the paw volume means were significantly higher for all groups compared to the initial paw volumes (Figure 3). The groups treated with diclofenac p.o and topic (NSAID p.o and NSAID topic) had the lowest increase in edema compared to the group treated with saline solution. At 24 h, the BOE groups (p.o and topic) did not have significantly lower values than the Control group, as was observed for the NSAID groups (p.o and topic). At 72 h, we observed a shift for the BOE topic group with a significant reduction in paw volumes compared to the Control group but at higher values compared to NSAID groups. At 168 h, the BOE groups (p.o and topic) had significantly reduced paw volumes compared to the Control group (Supplementary Table S2).

### 2.3. Pain Assessment

#### 2.3.1. Antinociceptive Effect on Acute Inflammation

The analgesic effect of BOE was tested using mechanical-induced pain on the inflamed paw. A significant antinociceptive effect was observed only at a 90-min time point for the oral NSAID group (Table 2). Both NSAID and BOE topical administration did not improve the pain perception at the 90-min time point. No antinociceptive effect of NSAIDs and BOE in either oral or topical administration was observed at 180, 240, and 360-min time intervals compared to the Control group.

#### 2.3.2. Antinociceptive Effect in Subacute Inflammation

In the second part of the experiment, we evaluated the antinociceptive effect of BOE in oral and topic administration in comparison to the Control and NSAID groups on adjuvant Freud-induced paw inflammation. The threshold values were not statistically different between groups at all time points except for the 72 h measurements. The pain threshold was significantly lower in the groups treated with a topical administration of BOE and NSAIDs vs. the Control group (Table 3).

### 2.4. Assessment of Oxidative Stress in Subacute Inflammation

**Figure 4:**
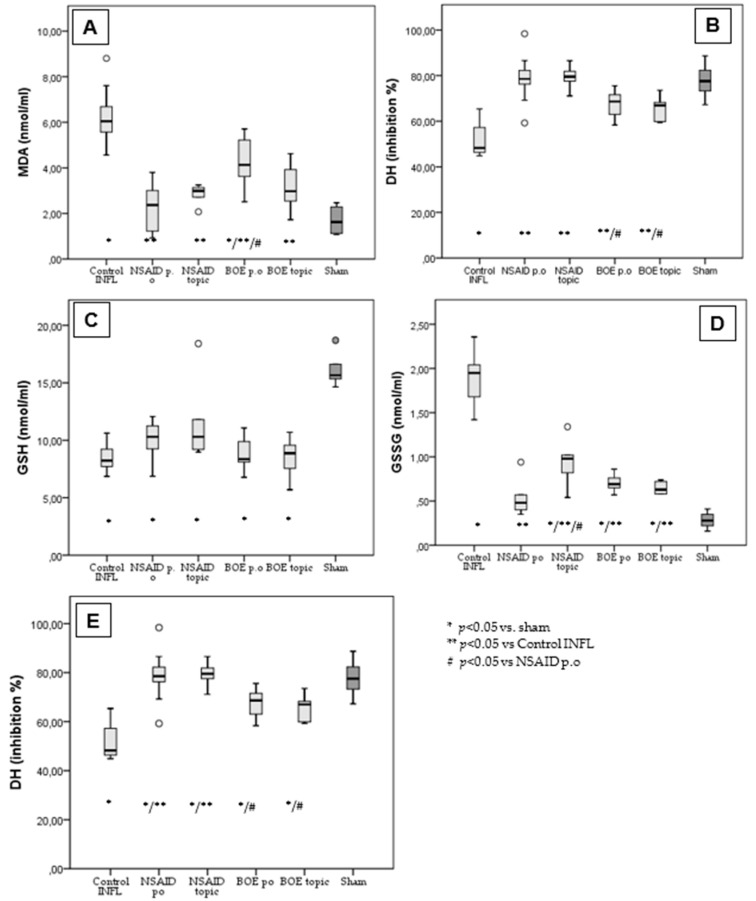
BOE’s effect on oxidative stress in subacute inflammation. (**A**): Malondialdehyde measurement in treated groups vs. Control and sham. (**B**) Hydrogen donor capacity in treated groups vs. Control and sham. (**C**) Glutathione in treated groups vs. Control and sham. (**D**) Oxidized glutathione in treated groups vs. Control and sham. (**E**) Superoxide dismutase in treated groups vs. Control and sham (MDA—malondialdehyde, GSH—glutathione, GSSG—oxidized glutathione, DH—hydrogen donor capacity, SOD—superoxide dismutase, BOE—*Brassica oleracea* extract, Control INFL—group with Freund’s adjuvant induced inflammation and no treatment, NSAID—nonsteroidal anti-inflammatory drug, p.o—oral treatment).

FA induced considerable systemic inflammation, increasing the serum levels of MDA and GSSG and decreasing those of GSH, DH and SOD compared to the sham. The NSAID p.o group, used as a positive control of an anti-inflammatory effect, limited the increase in MDA and GSSG and decreased DH and SOD. Malondialdehyde (MDA) in the Control group with inflammation is significantly higher (6.28 ± 1.2 nmol/mL) compared to all measured groups and compared to the historical sham group (1.7 ± 0.59 nmol/mL). The mean values of MDA in the groups treated with NSAID p.o (2.26 ± 0.97 nmol/mL), NSAID topic (2.82 ± 0.46 nmol/mL) and BOE topic (3.12 ± 1.02 nmol/mL) were not significantly different to the sham group except for the group with BOE p.o (4.29 ± 1.15 nmol/mL) (Figure 4A).

Hydrogen donor capacity (DH mean% of inhibition) was significantly higher in all treated groups, including NSAID p.o (78.57 ± 10.25%), NSAID topic (79.32 ± 5.69%), BOE p.o (67.39 ± 6.5%) and BOE topic (65.82 ± 5.4%) compared to the Control group (51.17 ± 6.75%) and comparable with the sham group (77.7 ± 7.5%). The NSAID groups had a significantly higher DH than the BOE groups (Figure 4B).

The glutathione (GSH) mean values in all groups (Control—8.37 ± 1.1 nmol/mL; NSAID p.o 9.94 ± 1.55 nmol/mL; NSAID topic 11.7 ± 3.89 nmol/mL; BOE p.o 8.88 ± 1.47 nmol/mL; BOE topic 8.54 ± 1.73 nmol/mL) are significantly reduced compared to the sham group (16.11 ± 1.42 nmol/mL) (Figure 4C).

For oxidized glutathione (GSSG), all treated groups (NSAID p.o 0.51 ± 0.17 nmol/mL; NSAID topic 0.94 ± 0.29 nmol/mL; BOE p.o 0.7 ± 0.09 nmol/mL; BOE topic 0.64 ± 0.06 nmol/mL) had significantly lower mean values compared to the Control group (1.9 ± 0.28 nmol/mL) but higher values than the sham group (0.28 ± 0.09 nmol/mL) (Figure 4D).

The mean values of superoxide dismutase (SOD) are lower for all treated groups (NSAID p.o 1290.49 ± 181.56 UI/L; NSAID topic 1174 ± 78.7 UI/L; BOE p.o 939.6 ± 79.8 UI/L; BOE topic 1132.7 ± 135.8 UI/L) compared to the sham group (1633.12 ± 155.5 UI/L) but to a lesser extent compared to the Control group (928.6 ± 101 UI/L). There are also significant differences between the NSAID p.o treated and BOE-treated groups.

## 3. Discussion

The present study analyzed the composition of a white cabbage variety from Romania, showing its content in antioxidants, and confirmed the efficacy of BOE extract in reducing inflammation and oxidative stress in experimental models of acute and subacute inflammation in female rats.

The phytochemical analysis of BOE included the determination of total polyphenol content and antioxidant activity. The total polyphenols content of the Romanian white cabbage variety was approximately 1.5-fold higher than the TPC of the white cabbage cultivar from Croatia [22], having approximately the same concentration as the Irish white cabbage (86.4 mg GAE/100 g fresh weight) [23].

FTIR is a method for the preliminary characterization of the molecular structure of different chemical compounds of plant extracts, which are complex mixtures. FTIR analysis showed the specific absorption pattern of the brassica species [24].

The LC-MS analysis of BOE identifies four compounds belonging to glucosinolates and their metabolites. Glucosinolates found in cruciferous vegetables like white cabbage are initially broken down into various compounds, including isothiocyanates, by myrosinase during food preparation, chewing or digesting, in the mouth and stomach, by the plant myrosinase and in the intestine, by the bacterial myrosinases [25]. Once formed, isothiocyanates can undergo further metabolism in the liver, so secondary metabolites are obtained. Thus, the oral administration of cabbage may provide active ingredients that are converted into components with different pharmacological activity which are delivered at the site of action. So, the final dynamic activity is different compared to topical administration when the active components cannot be converted into other active substances. The ethanolic extract of *Brassica oleracea* contained mainly metabolites. Probably the grinding and ethanol extraction process influences this transformation. In the traditional medicine in Romania, the cabbage leaves are smashed before use, probably activating plant myrosinase in this way, and sometimes alcohol is added to the smashed leaves. This is the reason we used cold ethanolic extract in our study.

Glucosinolates and their metabolites, isothiocyanates, are the most studied for their potential anti-inflammatory effects due to their ability to inhibit certain inflammatory pathways. Sulforaphane, an isothiocyanate present in cruciferous vegetables, induces the Nrf2 pathway and inhibits NF-κB contributing to anti-inflammatory and antioxidant responses [26]. Sulforaphane has a covalent interaction with the -SH functional group to induce its diverse functions in small molecules such as glutathione and in different enzymes [27]. The flavonols identified in the BOE extract may have antioxidant and anti-inflammatory effects. Kaempferol, a high lipophilic flavonol with a good bioavailability, has a significant inhibitory effect on cyclooxygenase 1 and 2, reducing inflammation [28]. Other components from the hydroxycinnamic acid derivatives identified in BOE with proven anti-inflammatory and anti-oxidant effects are para-coumaric acid [29], which acts by decreasing the cell-mediated immune response and the expression of the inflammatory mediators TNF-*α* and IL-6 and caffeoylquinic acid, which acts by downregulating the NRF2/HO-1 inflammation pathway [30].

Rokayya et al. evaluated the anti-inflammatory and antioxidant potential of cabbage preparation in vitro [31], while others have continued this research in in vivo animal studies [3,5]. *Brassicaceae* acted in vivo mainly on the NFκβ pathway, modulating the expression of inflammatory mediators and cytokines. In our study, BOE in topical administration showed an anti-inflammatory effect comparable to oral or topical NSAIDs. The oral administration of BOE did not reduce inflammation relative to the Control group. Sulforaphane and other active isothiocyanates are likely to be metabolized in the intestine and liver, so the effect may be reduced after oral administration, but other components may have a good bioavailability to induce in vivo effects if they are used in therapeutic concentrations.

The anti-inflammatory effects of dietary components after oral administration may vary depending on which conditions and specific inflammatory pathways are studied. There are also differences related to the metabolism of cabbage and species. Animal studies demonstrated an anti-inflammatory effect of cabbage extract in topical administration on a mouse model of contact dermatitis [4], but also the oral administration of it showed a reduction in inflammatory markers in a non-alcoholic fatty liver disease rat model [32]. Lee et al. demonstrated reduced immune cell infiltration and pro-inflammatory cytokines in inflamed tissues [4]. Kasarello et al. raised the hypothesis that the plant-encoded nucleic acid sequence specifically targets a human gene involved in inflammatory response, and they demonstrated that miRNA172a from *Brassica oleracea* reduces the expression of the FAN protein in human cells, thus lowering the pro-inflammatory activity of TNF-α [33]. So, bioactive components of *Brassica* species can modulate the expression of pro-inflammatory genes and reduce the production of inflammatory cytokines, such as interleukin-6 (IL-6) and tumor necrosis factor-alpha (TNF-alpha). Modulatory positive effects of *Brassica* ointment on the repair of skin wounds were also demonstrated [6].

Although a significant anti-inflammatory effect was observed in our study, we cannot assert the same conclusion about pain resolution. Peripheral and central mechanisms are involved in producing the analgesic effect, and it is possible that those active compounds that cause local effects are not in a sufficient concentration in the blood or do not have a good distribution into the body to have a systemic analgesic effect. The components of inflammation, edema, and hypersensitivity do not necessarily evolve in the same way at the same speed. For example, glutamate or calcium channel antagonists can reverse inflammatory hypersensitivity without ameliorating the edema [34] or may reduce edema without reducing the pain by vasoconstrictors. The increased pain observed at 90 min with topical application of substances, compared to the Control group, can be explained by the fact that local application is a maneuver that can cause increased pain. Subsequently, this effect was not again observed. Also, the adaptive mechanisms associated with pain may produce a peripheral sensitization of the nociceptors which is processed at the central level, that can exacerbate pain responses, lower pain threshold, and produce persistent pain [35]. We observed this effect in the acute setting and at 24 h in the subacute model for the Control group. Therefore, we considered it important to evaluate by comparison of all groups, not only concerning the initial state in each group. Due to the increased dispersion of values at the algesimeter in the Control group between different time points, we consider that the data cannot be interpreted in one way or another. We cannot draw relevant conclusions regarding the analgesic effect of BOE.

The antioxidant effect of BOE extract was assessed by measuring the serum values for MDA, GSH, GSSG, SO, DH, and reduced lipid peroxidation and NOx production, which was demonstrated in our study. MDA is a marker of oxidative stress that is generated in vivo through prostaglandin synthesis or lipid peroxidation. Studies have shown that NSAIDs may reduce MDA production but, because of the heterogeneity in the chemical structure of NSAIDs, the effect may be different. Diclofenac, which we used in our study as an NSAID, has an antioxidant effect by scavenging some of the stable free radicals responsible for the propagation phase without having a radical-trapping effect for superoxide anion (O^2−^) and hydroxyl radicals (**·**OH) [36]. The high content of isothiocyanates influenced glutathione levels by the covalent -SH interaction [27]. In our study, treatment with NSAIDS and topical BOE prevented the increase in oxidative stress markers induced by inflammation. We may assume that the topical administration of BOE may reduce locally the oxidative stress and thus the inflammatory process, while the oral administration of it requires a longer duration of therapy to obtain the same beneficial effects. While isothiocyanates, mainly sulforaphane from white cabbage and other cruciferous vegetables, are known to have anti-inflammatory potential [27], more research is needed to fully understand the mechanisms of other components and the extent of their anti-inflammatory properties.

The strength of this study consists of its complex evaluation of the *Brassica oleracea* composition and its anti-inflammatory, analgesic, and antioxidative effects in different models of acute or subacute inflammation and different ways of administration. Documented research supports the traditional use of cabbage in improving inflammation and inflammatory pain. It is the first analysis of the Romanian cultivar of *Brassica oleracea* composition. The study has also some limitations. The authors investigated the effects of ethanolic extract of *Brassica oleracea* but not the effects of bioactive compounds that may confer particular pharmacological properties. The analgesic and anti-inflammatory effects of *Brassica oleracea* in acute and subacute inflammation were investigated using both oral and topical administration of the extract, and the anti-inflammatory effect was confirmed for the topical use. Our results also support the idea of evaluating the effects after a longer administration on the oral route in subacute inflammation, which might increase the intensity and duration of its pharmacological effects.

In conclusion, the obtained data may confirm the anti-inflammatory and antioxidative effect of *Brassica oleracea* in topical administration and partially in systemic administration in an induced inflammation in female rat models. The therapeutic effects of *Brassica oleracea* could be a consequence of the isothiocyanates content of the cabbage species from Romania.

## 4. Materials and Methods

### 4.1. Plant Material and Extraction

*Brassica oleracea var. capitata f. alba* (common cabbage, the green version) originated from Cluj County and was purchased from the local market in June at commercial maturity. Heads were cleaned (1200 g) and cut into small pieces and squeezed out of the containing water, which was separately collected. The remaining cabbage was macerated with 1200 mL of ethanol for 24 h at 4 °C and finally sonicated at room temperature for 30 min. The extract was then filtered using filter paper and then combined with the previously obtained water extract and evaporated using a vacuum evaporator (Hei-VAP Platinum 3) until we obtained a volume of 400 mL of extract.

### 4.2. Analysis of Basic Constituents of Brassica oleracea Extract (BOE)

The total phenolic content (TPC) determination was estimated spectrophotometrically using the Folin–Ciocalteu reagent as previously described [37]. To the 25 μL of BOE extract was added 125 mL Folin–Ciocalteu reagent (0.2 N) and 100 mL solution of sodium carbonate (Na_2_CO_3_), 7.5% (*w*/*v*). The mixture was allowed to stand for 2 h in dark conditions, and absorption was measured at 760 nm using a Synergy HT Multi-Detection Microplate Reader (BioTek Instruments, Inc., Winooski, VT, USA). Results were expressed as mg gallic acid equivalents (GAE), consisting in mg/100 g fresh weight of extracts. Measurements were performed in triplicate.

### 4.3. FTIR Analysis

Fourier-transform infrared (FT-IR) measurements were performed using an IR Prestige-21 spectrometer (Shimadzu, Japan) with attenuated total reflectance (ATR) and an internal reflection accessory of zinc selenide composite. The BOE FTIR spectra were measured directly after being pipetted on the ZnSe ATR crystal. The measurement range was set between 4000 and 650 cm^−1^, and 64 scans were performed. Water was used to obtain the background spectra. Acetone was used to clean the ATR crystal between measurements.

### 4.4. Liquid Chromatography-Diode Array Detection–Electro-Spray Ionization Mass Spectrometry (HPLC-DAD-ESI MS) Analysis

The HPLC-MS analysis of BOE extract was performed according to the methods described by Pop et al. [37]. We used an Agilent 1200 HPLC equipped with a DAD detector and coupled to a 6110 Agilent single quadrupole mass spectrometer (MS) to analyze the extract. The compounds extracted in the BOE extract were separated on a 4.6 mm × 150 mm, 5 µm Eclipse XDB C18 column (Agilent Technologies, Santa Clara, CA, USA). The separation took place at room temperature using 0.1% acetic acid/water (99:1) (*v*/*v*) as mobile phase A and 0.1% acetic acid/acetonitrile (99:1) (*v*/*v*) as mobile phase B. The elution gradient (expressed as % B) is 0 min, 5% B; 0–2 min, 5% B; 2–18 min, 5–40% B; 18–20 min, 40–90% B; 20–24 min, 90% B; 24–25 min, 90–5% B; 25–30 min, 5% B. The flow was set at 0.5 mL/min, and the spectra were registered between 200 and 600 nm. The MS scan was performed between 100 and 1200 *m*/*z* in the ESI+ mode. The compounds fragmentation had the following ionization conditions: 300 °C, a capillary voltage of 3000 V, and a nitrogen gas flow of 7 L/min. Data analysis was performed using the Agilent ChemStation Software version B.04.03 (Rev B.04.02 SP1, Palo Alto, CA, USA). Interpretation was performed considering the UV-visible spectra, the retention time, and mass spectra information as well as comparison with authentic standards (when available). The reproducibility and precision of the HPLC analysis were performed using the calibration curve of chlorogenic acid (y = 22.585x − 36.728, (R^2^ = 0.9937), LOD = 0.41 μg/mL, LOQ = 1.24 μg/mL), of luteolin (y = 68.857x + 25.113, (R^2^ = 0.9972), LOD = 0.38 μg/mL, LOQ = 1.14 μg/mL) and of rutin (y = 26.935x − 33.784, (R^2^ = 0.9981), LOD = 0.21 μg/mL, LOQ = 0.64 μg/mL).

### 4.5. Animals

Fifty female Wistar rats (weighing 150–230 g) were obtained from the Biobase of Iuliu Hațieganu University of Medicine and Pharmacy Cluj-Napoca. The animals were housed in cages, ten rats/cage, with wood shavings in the bottom, at 22 C, and for a 12 h light/dark cycle, with a standard pellet diet and water ad libitum. The animals were maintained following the recommendations of the Guide for the Care and Use of Laboratory Animals, and the protocol was approved by the Ethics Committee of Iuliu Hațieganu University of Medicine and Pharmacy and by the Sanitary-Veterinary and Food Safety Directorate from Cluj-Napoca (no 61/08.05.17). The animals were randomly divided into five groups. To reduce the use of animals in the experimental studies, the same animals were used for both models of inflammation with a 2-week recovery period between the carrageenan-induced inflammation and Freund’s adjuvant-induced inflammation, changing also the affected paw: right posterior paw first, left posterior paw second.

### 4.6. Induction of Inflammation and Experimental Design

#### 4.6.1. Carrageenan-Induced Paw Inflammation in Rats

The acute inflammation was induced using the experimental model of carrageenan-induced paw edema [34]. Each rat was injected with 0.1 mL of 1% carrageenan suspension freshly prepared (Sigma- Aldrich, St. Louis, MO, USA) into the sub-plantar region of the right posterior paw.

#### 4.6.2. Freund’s Adjuvant-Induced Inflammation

The subacute inflammation was induced using the experimental model of Freund’s adjuvant-induced inflammation [34]. Each rat was injected with 0.1 mL of complete Freund’s adjuvant solution in the left posterior paw (sub-plantar region).

#### 4.6.3. Routes of Administration and Rhythm of Administration

For these experiments, we chose two different routes of administration of the substances, oral and topic. The groups, the routes of administration and doses are described in Table 4. Orally, the substances were given by gavage. For the topical administration, we applied BOE, NSAID or saline solution directly on the inflamed paw with 10 s of light massage to ensure appropriate diffusion on the skin.

**Figure 5:**
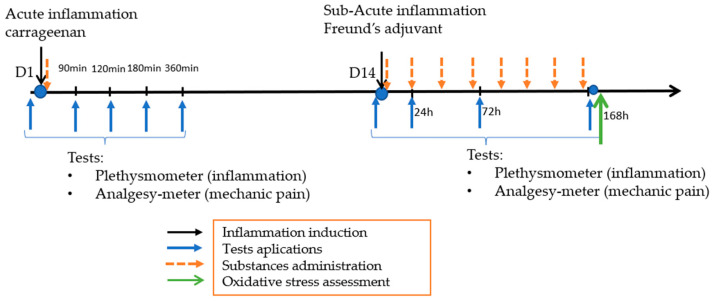
Experiments timeline.

For the first part of the experiment, carrageenan-induced inflammation, we used only one dose of tested substances right after the carrageenan injection. In the second part, the substances were administered for 7 consecutive days, starting after Freund’s adjuvant administration (Figure 5).

### 4.7. Experimental Tests

#### 4.7.1. Timeline

In the acute experiment, the initial evaluations were assessed 60 min before carrageenan injection and at 90, 120, 180, and 360 min after carrageenan injection. The tested substances were administered immediately after the carrageenan injection. In the subacute experiment, the initial tests were assessed 60 min before injection of Freund’s adjuvant and at 24, 72, and 168 h after it (Figure 5).

#### 4.7.2. Inflammation Assessment

We used a digital plethysmometer by Ugo-Basile (Milan, Italy) to measure the paw edema by recording the volume of fluid displaced by the affected paw. The results were expressed as paw volume. We calculated the relative paw volume expansion using the ratio between the detected and initial paw volume for each rat.

#### 4.7.3. Pain Assessment

Mechanical test: The mechanical evaluation of acute inflammation-induced pain was assessed using Randall–Selitto paw-pressure test (Analgesy-Meter Ugo Basile, Milan, Italy). Plantar mechanical pressure was applied linearly by increasing the mechanical force on the rat’s paw. The applied pressure (measured in grams) was recorded as the baseline threshold before carrageenan injection and as the paw withdrawal threshold for time intervals established in the protocol. The value of 250 g was set as a cut-off pressure to reduce the harm to the animal. A percentage of the maximal possible analgesic effect was calculated setting the value of 250 g as maximum.

All the tests were performed in a blinded manner by the same investigator (O.S), who was different from the one that administered the substances (R.M.P).

#### 4.7.4. Oxidative Stress Assessment

At the end of the experimental period, blood samples were collected on the anticoagulant collection tube, using a thick capillary, from the retro-orbital plexus. The serum samples obtained after centrifugation were stored at −80 °C until they were analyzed. Serum oxidative stress status was evaluated using previously described spectrophotometric methods for the following parameters: malondialdehyde (MDA) [38], glutathione (GSH) [39], oxidized glutathione (GSSG) [40], hydrogen donor capacity (DH) [41], and superoxide dismutase (SOD) [42]. To compare the values obtained in our experiment, we used a sham comparator group with no inflammation to reduce animal use. These data were acquired from our research team using the same methods for the oxidative stress assessment and the same conditions for the animals.

#### 4.7.5. Chemicals

Complete Freund’s adjuvant and carrageenan were purchased from Sigma–Aldrich (St. Louis, MO, USA). Diclofenac solution, diclofenac gel, and saline solution were purchased from a local pharmacy.

#### 4.7.6. Statistical Analysis

The statistical analysis was performed using SPSS version 18 (Chicago, IL, USA). The results were expressed as mean and deviation (for variables with normal distribution). We used the one-way ANOVA test with Tukey correction and the ANOVA test for repeated measures. The level of statistical significance was set at *p* < 0.05.

## Figures and Tables

**Table 1:** LC-MS phenolic compound tentative identification from BOE.

No.	Rt (min)	Lambda Max (nm)	[M + H]^+^ *m*/*z*	Tentative Identification	References
1	3.1	220, 280, 350sh	380	Desulfo-glucoraphanin	[15]
2	4.7	220, 270	308	Hydroxycinnamic acid para coumaric acid	[10]
3	8.1	260	422	Glucoerucin	[16]
4	8.9	275	354/355	Caffeoylquinic acid	[10]
5	12.2	225, 310	386	Synapoyl glucoside acid	[10]
6	13.2	220, 240sh, 275, 325	433	Apigenin-glucoside	[17]
7	13.6	220, 275	433	Kaempherol rhamnoside	[18]
8	14.2	220, 280, 290, 315sh	521	Isorhamnetin 3-o acetyl glucoside	[19]
9	14.7	220, 250, 315	565	Apigenin-apiosyl-glucoside	[18]
10	15.8	220, 280	437	Glucoraphanin	[20]
11	16.5	280, 320sh	569	Quercetin 3-o-6-benzoyl-galactoside	[21]
12	18.2	225, 80	391	Desulfo-glucobrassicin	[15]

**Table 2:** The effect on induced mechanical pain in the acute inflammation model.

Time	Control	NSAID p.o	BOE p.o	NSAID Topic	BOE Topic	*p*
0 min	9.65 ± 2.69 (38.6%)	9.8 ± 2.58 (39.2%)	9.40 ± 7.05 (37.6%)	9.45 ± 4.92 (37.8%)	9.15 ± 4.6 (36.6%)	NS
90 min	9.1 ± 1.83 (34.4%)	11.60 ± 4.14 (46.4%) *	7.45 ± 3.20 (29.8%)	5.55 ± 1.32 (22.2%) *^/^**	5.9 ± 1.94 (23.6%) *^/^**	<0.001
180 min	7.4 ± 1.71 (29.6%)	10.27 ± 2.98 (41.1%)	8.57 ± 2.88 (34.3%)	8.60 ± 2.47 (34.4%)	10.7 ± 6.68 (42.8%)	NS
270 min	7.92 ± 2.73 (31.7%)	9.15 ± 3.21 (36.6%)	9.63 ± 3.37 (38.5%)	7.50 ± 3.71 (30%)	8.7 ± 5.9(34.8%)	NS
360 min	6.9 ± 2.06 (27.6%)	7.6 ± 2.53 (30.4%)	6.35 ± 1.92 (25.4%)	6.00 ± 1.84 (24%) **	6.2 ± 1.49 (24.8%) **	NS

* Significance vs. the Control group (Control) (*p* < 0.05, analyzed post hoc Tukey correction). ** significance vs. positive Control group (NSAID p.o) (*p* < 0.05, analyzed post hoc Tukey correction). Data are expressed as mean ± SD and % from maximal 250 g pressure. NS not significative.

**Table 3:** The effect of BOE mechanical pain induced in subacute inflammation model.

Time	Control	NSAID p.o	BOE p.o	NSAID Topic	BOE Topic	*p*
0 h	7.3 ± 2.68	6.8 ± 2.86	5.3 ± 1.15	6.95 ± 1.75	5 ± 1.2	NS
24 h	4.75 ± 2.4	5.55 ± 1.12	5.8 ± 1.03	5.45 ± 1.03	5.5 ± 2.73	NS
72 h	8 ± 1.56	7.25 ± 1.37	7.35 ± 2.67	6.2 ± 1.82 *	5.2 ± 1.97 *	0.02
168 h	7.9 ± 2.41	5.25 ± 1.7	6.55 ± 1.48	6.16 ± 0.76	5.85 ± 1.47	NS

* Significance vs. the Control group (Control) (*p* < 0.05, analyzed post hoc Tukey correction). Data are expressed as mean ± SD and % from maximal 250 g pressure. NS not significative.

**Table 4:** Experimental design, routes, and used doses.

Groups/Abbrev.	Administrated Substances/Dose		Route
	Acute Inflammation—Single Dose	Subacute Inflammation—Daily Doses, 1 Week Treatment	
Group 1—Control inflammation	Normal saline solution	Normal saline solution	topic
Group 2—NSAID p.o	Diclofenac (10 mg/kg BW)	Diclofenac (5 mg/kg BW)	p.o
Group 3—BOE p.o	BOE (4 mL/kg BW)	BOE (4 mL/kg BW)	p.o
Group 4—NSAID topic	Diclofenac 0.5 mL gel 5%	Diclofenac 0.5 mL gel 5%	topic
Group 5—BOE topic	BOE 0.5 mL BOE	BOE 0.5 mL	topic

Abbreviations: BW, body weight; BOE, *Brassica oleracea* extract; p.o, per os.

## Data Availability

The original contributions presented in the study are included in the article (and Supplementary Material), further inquiries can be directed to the corresponding authors.

## Supplementary Materials

The following supporting information can be downloaded at: https://www.mdpi.com/article/10.3390/molecules29153448/s1. Table S1. The effect of BOE on acute inflammation; Table S2. The effect of Brassica extract on subacute inflammation.
