# Supplementary material for: The Anti-Inflammatory, Analgesic, and Antioxidant Effects of Polyphenols from Brassica oleracea var. capitata Extract on Induced Inflammation in Rodents
Source: Molecules. 2024 Jul 23;29(15):3448. doi: 10.3390/molecules29153448 (PMC11313733; doi:10.3390/molecules29153448)
Supplement: Supplementary file 1 [file molecules-29-03448-s001.zip › molecules-3094903-supplementary.pdf]

Table 1 The effect of BOE on acute inflammation

Time point	Paw volume (ml) Mean±SD<br>Relative paw volume increase (Mean±SD)					
	Control	NSAID p.o	BOE p.o	NSAID topic	BOE topic	p
0 min	2.13±0.31	2.47±0.25	2.41±0.28	2.67±0.21	2.85±0.31	NS
90 min	3.67±0.26<br>(74.43±13.42)	2.64±0.26<br>(7.10±1.77)*	3.98±0.39<br>(68.32±31.72)**	3.01±0.26<br>(12.81±7.14)*	3.19±0.32<br>(12.65±11.55)*	<0.001
180 min	4.04±0.35<br>(93.21±30.71)	2.68±0.31<br>(8.47±6.18)*	4.49±0.37<br>(89.60±31.73)**	3.04±0.35<br>(14.26±13.0)*	3.54±0.29<br>(25.06±12.8)*	<0.001
270 min	4.12±0.20<br>(97.18±27.34)	2.97±0.30<br>(20.20±6.6)*	4.43±0.33<br>(85.72±20.02)**	3.48±0.46<br>(30.33±13.73)*	3.82±0.35<br>(34.68±11.0)*	<0.001
360 min	4.00±0.28<br>(91.97±27.34)	3.36±0.47<br>(35.55±10.51)*	4.21±0.36<br>(76.56±18.19)**	3.50±0.49<br>(31.31±17.94)*	3.20±0.26<br>(12.81±9.22)*	<0.001

*significance vs control group (Control) (p<0.05, post-hoc analysis with Tukey correction)
**significance vs positive control group (NSAID p.o) (p<0.05 post-hoc analysis with Tukey correction)

Table2: The effect of Brassica extract on subacute inflammation

Time point	Paw volume (ml) Mean±SD<br>Relative paw volume increase (Mean±SD)					
	Control	NSAID p.o	NSAID topic	BOE p.o	BOE topic	p
0 h	3.57±0.35	3.92±0.43	3.73±0.41	3.38±0.62	3.58±0.30	NS
24 h	7.33±0.61<br>(106.7±23.86)	6.26±0.72<br>(59.41±9.8)*	6.16±0.85<br>(65.57±18.83)*	6.15±0.83<br>(85.45±31.26)**	6,51±0.75<br>(83.19±28.19)**	<0.001
72 h	7.40±1.07<br>(108.32±31.96)	4.92±0.78<br>(26.25±22.4)*	4.68±0.74<br>(25.36±15.22)*	6.58±0.82<br>(97.50±22.79)**	6.00±0.92<br>(67.62±21.55)*/**	<0.001
168 h	6.7±1.37<br>(88.38±38.83)	4.95±0.86<br>(27.83±29.68)*	4.51±0.67<br>(20.75±9.9)*	5.24±1.00<br>(56.44±23.14)*/**	5.26±0.76<br>(47.51±21.77)*/**	<0.001

*significance vs control group (Control) (p<0.05, post-hoc analysis with Tukey correction)
**significance vs positive control group (NSAID p.o) (p<0.05 post-hoc analysis with Tukey correction)
